# CLEC14A deficiency exacerbates neuronal loss by increasing blood-brain barrier permeability and inflammation

**DOI:** 10.1186/s12974-020-1727-6

**Published:** 2020-02-04

**Authors:** Yeomyeong Kim, Sungwoon Lee, Haiying Zhang, Sunghye Lee, Hyejeong Kim, Yeaji Kim, Moo-Ho Won, Young-Myeong Kim, Young-Guen Kwon

**Affiliations:** 1 Department of Biochemistry, College of Life Science and Biotechnology, Yonsei University, Seoul, 03722 South Korea; 2 Department of Neurobiology, School of Medicine, Kangwon National University, Chuncheon, 24341 South Korea; 3 Department of Molecular and Cellular Biochemistry, School of Medicine, Kangwon National University, Chuncheon, 24341 South Korea

**Keywords:** C-type lectin family 14 member A (CLEC14A), Blood-brain barrier (BBB), Ischemic stroke, Vascular endothelial growth factor (VEGF), Vascular endothelial growth factor receptor-2 (VEGFR-2)

## Abstract

**Background:**

Ischemic stroke is a main cause of mortality. Blood-brain barrier (BBB) breakdown appears to play a critical role in inflammation in patients with ischemic stroke and acceleration of brain injury. The BBB has a protective function and is composed of endothelial cells, pericytes, and astrocytes. In ischemic stroke treatments, regulation of vascular endothelial growth factor (VEGF)-A and vascular endothelial growth factor receptor (VEGFR)-2 is a crucial target despite adverse effects. Our previous study found that loss of C-type lectin family 14 member A (CLEC14A) activated VEGF-A/VEGFR-2 signaling in developmental and tumoral angiogenesis. Here, we evaluate the effects of BBB impairment caused by CLEC14A deficiency in ischemia-reperfusion injury.

**Methods:**

In vitro fluorescein isothiocyanate (FITC)-dextran permeability, transendothelial electrical resistance (TEER) assay, and immunostaining were used to evaluate endothelial integrity. BBB permeability was assessed using Evans blue dye and FITC-dextran injection in *Clec14a*^−/−^ (CLEC14A-KO) mice and wild-type mice. Middle cerebral artery occlusion surgery and behavioral assessments were performed to evaluate the neurologic damage. The change of tight junctional proteins, adhesion molecules, pro-inflammatory cytokines, and microglial were confirmed by immunofluorescence staining, Western blotting, and quantitative reverse transcription polymerase chain reaction of brain samples.

**Results:**

In endothelial cells, knockdown of CLEC14A increased FITC-dextran permeability and decreased transendothelial electrical resistance; the severity of this effect increased with VEGF treatment. Immunofluorescence staining revealed that tight junctional proteins were attenuated in the CLEC14A knockdown endothelial cells. Consistent with the in vitro results, CLEC14A-KO mice that were injected with Evans blue dye had cerebral vascular leakage at postnatal day 8; wild-type mice had no leakage. We used a middle cerebral artery occlusion model and found that CLEC14A-KO mice had severe infarcted brain and neurological deficits with upregulated VEGFR-2 expression. FITC-dextran leakage was present in CLEC14A-KO mice after ischemia-reperfusion, and the numbers of tight junctional molecules were significantly decreased. Loss of CLEC14A increased the pro-inflammatory response through adhesion molecule expression, and glial cells were activated.

**Conclusions:**

These results suggest that activation of VEGFR-2 in CLEC14A-KO mice aggravates ischemic stroke by exacerbating cerebral vascular leakage and increasing neuronal inflammation after ischemia-reperfusion injury.

## Background

Stroke is one of the most devastating neurological diseases and a major cause of long-term disability. Approximately 80% of strokes are ischemic [1, 2]. The standard treatment for stroke is thrombolysis, which has rather limited efficacy [3, 4]. Ischemic stroke induces blood-brain barrier (BBB) breakdown that contributes to secondary brain injury by causing cerebral edema, increasing hemorrhage, and exacerbating the inflammatory response [5, 6]. The associated damage aggravates ischemic stroke injury and post-stroke deficits by decreasing the capability of brain repair. The BBB has an important protective function [6]. It includes a basement membrane, endothelial cells (ECs), pericytes, and astrocyte end-feet that regulate the movement of molecules into the brain [7]. Tight junctional proteins (e.g., Occludin, ZO-1, and Claudin-5) connect brain ECs to form the BBB [8]. Targeting the BBB may be a promising therapeutic approach for the treatment of ischemic stroke.

Vascular endothelial growth factor (VEGF)-A is a crucial mediator of cerebral angiogenesis, and VEGF signaling pathways are potential therapeutic targets for stroke [9]. The VEGF-A/vascular endothelial growth factor receptor (VEGFR)-2 signaling pathway is essential for recovery from cerebral damage after ischemic stroke [10, 11]. VEGFs likely have multifaceted actions in stroke prevention and therapy [12]. Despite the potential adverse effects of VEGFs (e.g., vascular permeability and brain edema), the VEGF-A/VEGFR-2 signaling pathway has been used for acute or chronic treatment of stroke [9]. However, the effects of hyperactivation of VEGFR-2 in stroke are not fully understood.

The C-type lectin family 14 member A (CLEC14A) type I transmembrane protein belongs to the C-type lectin superfamily and includes an EGF-like domain along with the C-type lectin domain [13]. The carbohydrate-binding protein C-type lectin domain mediates functions, including inflammatory responses and cell-to-cell adhesion [13, 14]. CLEC14A is specifically expressed in ECs in mouse and human vasculature [15]. Accumulating evidence indicates that CLEC14A is a crucial regulator of physiological and pathological responses [16, 17]. Studies of CLEC14A in angiogenesis (e.g., cell-to-cell adhesion, endothelial migration, and tube formation) have been reported [13, 18]. CLEC14A also regulates proangiogenic phenotypes in various carcinomas [16, 17, 19]. Our previous study found that CLEC14A interacts with VEGFR-3 and maintains VEGF-A-induced VEGFR-2 levels in developmental and tumoral angiogenesis [20]. In this study, we used CLEC14A knockout (KO) mice to evaluate the effects of BBB hyperpermeability caused by VEGFR-2 activation. We found that regulation of BBB permeability is critical for ischemia-reperfusion injury.

## Methods

### Cells

Collagenase type II (Worthington Biochemical Corporation, Lakewood, NJ) was used to isolate human umbilical vein endothelial cells (HUVECs) from umbilical cord veins. The cells were cultured on gelatin-coated plates in Medium 199 (Invitrogen, Carlsbad, CA; Thermo Fisher Scientific, Waltham, MA) supplemented with 20% fetal bovine serum, 100 U/ml penicillin-streptomycin, 3 ng/ml basic fibroblast growth factor (bFGF; R&D Systems), and 5 U/ml heparin. Trypsinization was used to collect the cells, which were then used for assays up to passage 7. Human brain microvascular endothelial cells (HBMECs; Applied Cell Biology Research Institute, Kirkland, WA) were used for assay up to passage 10. The HBMECs were cultured on gelatin-coated plates and maintained in endothelial cell basal medium-2 (CC-3156) contained in an endothelial cell growth medium-2 kit (CC-4176) (Clonetics, Lonza, Walkersville, MD) and in 20% fetal bovine serum.

### RNA interference

HUVECs and HBMECs were transfected for 3 h with non-targeting siRNA or CLEC14A siRNA using lipofectamine (Invitrogen, Thermo Fisher Scientific) and assayed 36 h after transfection. The sequence of the CLEC14A siRNA (5′-CAAUCAGGGUCGACGAGAA-3′) was designed by Dharmacon Inc. (Lafayette, CO).

### In vitro permeability assay

HUVECs and HBMECs were cultured on the luminal side of filters (0.4-μm pore size; Corning, NY) in gelatin-coated 12-well plates until confluent. They were then cultured in endothelial serum-free medium (i.e., serum-starved) for 2 h and exposed to VEGF-A (50 ng/ml) for 30 min. A Millicell ERS-2 V/ohm meter (Millipore, Billerica, MA) was used to measure the transendothelial electrical resistance (TEER). The results for the cell-free gelatin-coated filter TEERs were subtracted from the results for the measured TEERs, and the final results were presented in Ω × cm^2^ units. Fluorescein isothiocyanate (FITC)-dextran fluorescein was used to confirm the paracellular permeability associated with the TEER measurement. FITC-dextran (1 mg/ml; Sigma, St. Louis, MO) was added to the upper compartments of the transwell cultures. A FLUOstar Omega microplate reader was used to measure the values for absorbance of the lower chamber solution at 492 nm (excitation) and 520 nm (emission) wavelengths.

### Immunofluorescence staining of HUVECs and HBMECs

After fixation in 4% formaldehyde for 15 min at room temperature, the HUVECs and HBMECs were permeabilized in 0.1% Triton X-100 in phosphate-buffered saline (PBS) for 15 min at 4 °C. The cells were then exposed to antibodies [i.e., rat anti-VE-cadherin (1:200, Invitrogen), mouse anti-claudin-5 (1:50, Invitrogen), rabbit anti-occludin (1:100, Invitrogen), or rabbit anti-zonula occludens-1 (ZO-1, 1:200, Invitrogen)] overnight at 4 °C. Cells were then incubated (1 h) at room temperature with Alexa Fluor-conjugated secondary antibodies. To stain the nuclei, the cells were exposed to 4,6-diamidino-2-phenylindole (DAPI) diluted (1:500) in PBS during the washing step. Dako mounting reagent was used to mount the cells, and a confocal microscope (LSM 700 META, Carl Zeiss) was used to examine the results.

### Western blot analysis

Proteins from ischemic hemispheres were extracted in radioimmunoprecipitation assay (RIPA) buffer (100 mM Tris-Cl, 5 mM EDTA, 50 mM NaCl, 50 mM β-glycero-phosphate, 50 mM NaF, 0.1 mM Na_3_VO_4_, 0.5% NP-40, 1% Triton X-100, and 0.5% sodium deoxycholate). Sample protein concentration was quantified using a SMART BCA Protein Assay kit (iNtRON Biotechnology, Inc., Gyeonggi-do, Korea). Next, cell lysates (20 μg) were separated by sodium dodecyl sulfate-polyacrylamide gel electrophoresis and then transferred to nitrocellulose membranes. The membranes were blocked with 3% bovine serum albumin in 0.1% tris-buffered saline with Tween 20 (TBST) and then probed with primary antibodies. The membranes were then incubated with horseradish peroxidase-conjugated goat anti-rabbit IgG or goat anti-mouse IgG (Invitrogen) secondary antibodies. β-Actin was used as a loading control. The following primary antibodies were used at a 1:1000 dilution: CLEC14A (Santa Cruz Biotechnology Inc., Dallas, TX; sc-246,296), phospho-VEGFR2 (Cell Signaling Technology, Danvers, MA; 2478), VEGFR2 (Cell Signaling Technology; 2472), Claudin-5 (Invitrogen; 34-1600), Occludin (Invitrogen; 710,192), zona occluden-1 (ZO-1) (Invitrogen; 61-7300), and β-actin (Invitrogen; MA5-15739).

### Experimental animals

Each mouse (6 to 8 weeks of age) was maintained on a C57BL/6 background. The RPCI-22 Mouse 129S6/SvEvTAC Taconic BAC Library (https://bacpacresources.org) provided the CLEC14A clone. A Southern blot probe was synthesized to test the probe for embryonic stem cell screening. A CLEC14A-KO mouse construct was generated using a pgkTK vector. The CLEC14A-KO mice were produced in a BacVac cassette using homologous recombination of *Clec14a* and LacZ + Neo.

### Vascular permeability assay

Evans blue (EB; 2% in PBS; Sigma-Aldrich, E2129) dye and two different sizes of FITC-dextran (4 and 70 kD; Sigma-Aldrich, 46944 and 46945, respectively) were used to evaluate cerebrovascular leakage. To test wild-type (WT) and knockout (KO) mice under normal conditions, the EB dye was injected via the intraperitoneal route 24 h before the brain tissue was removed and FITC-dextran (4 kD) was injected into the left ventricle 30 min before the whole brain was collected. To perform the vascular leakage assay in the ischemic stroke mouse model, FITC-dextran (70 kD) was injected (30 mg/ml) into the left ventricle and EB dye was injected via the intravenous route 30 min before the mice were sacrificed. EB leakage was quantified in the brain tissue after the brain had been homogenized and incubated in formamide (24 h, 55 °C). The EB assay result was measured in the supernatant from each sample (absorbance, 620 nm). The EB concentrations were normalized based on the results for the sham-operated brain samples. The results were calculated using a standard curve of EB in formamide and were presented as microgram per gram of brain tissue.

### Cryosectioning and immunofluorescence staining

Brain tissue was exposed to paraformaldehyde (4%) and PBS (pH 7.4) overnight (4 °C) for fixation. The tissue was then rinsed with PBS at room temperature, incubated overnight (4 °C) in sucrose (15%), and then transferred to sucrose (30%) at 4 °C until the tissue sank. Tissue-Tek optimum cutting temperature (OCT) embedding medium was then used to infiltrate the fixed brains for 30 min at room temperature. They were stored at − 70 °C after transfer to an OCT-filled embedding mold and freezing with dry ice. While frozen, sections (20- to 40-μm-thick) were cut onto slides at − 20 °C for immunostaining. The slides were stored at − 70 °C until use for this procedure. Briefly, the sections were prefixed in acetone for 30 min at − 70 °C and briefly air dried. Flowing water was used to rinse the OCT. Each section was then exposed to blocking solution (1 h, 37 °C). The sections were then incubated overnight in primary antibody (1:500, 4 °C), washed three times (10 min per wash) with Triton X-100 (0.1%) in PBS, and incubated overnight in secondary antibody (1:500, 4 °C). The sections were then counterstained using DAPI (1 μg/ml) and washed five times with Triton X-100 (0.1%) in PBS (10 min per wash). Antibody diluent (Dako, Agilent Technologies, Santa Clara, CA) was used to dissolve each antibody. A confocal microscope (LSM 700 META, Carl Zeiss) was used to examine each section.

### Induction of transient focal cerebral ischemia

Anesthesia using a mixture of 2.5% isoflurane (Baxtor, Deerfield, IL) in 33% oxygen and 67% nitrous oxide was administered to each mouse via a facemask. Two percent isoflurane was used for the maintenance anesthesia. A rectal temperature probe was inserted, and a heating pad was used to maintain the body temperature (37 °C). Middle cerebral artery occlusion (right side) was used to induce focal cerebral ischemia [21]. Briefly, a midline cervical incision was used to expose the right common carotid artery. After the right external carotid artery was dissected free, it was isolated distally by coagulating its branches and placing a distal ligation prior to transection. A 6–0 fine middle cerebral artery occlusion (MCAO) suture (Doccol Corporation, Sharon, MA) was inserted into the lumen of the right external carotid artery stump. To occlude the ostium of the MCAO, the suture was carefully advanced into the internal carotid artery 8 mm from the bifurcation. The suture was removed after an hour of ischemia. After recovery and until euthanasia, each mouse was kept in a thermal incubator to maintain body temperature. The same surgical procedure was used for the sham-operated animals, but the middle cerebral artery was not occluded.

### Inhibition of VEGFR-2

Mice were subcutaneously injected with VEGFR-2 inhibitor (50 mg/kg), SU5416 (Tocris Bioscience, Bristol, GB), in solution (0.5% carboxy methyl cellulose, 0.9% sodium chloride, 0.4% polysorbate, 0.9% benzyl alcohol in dH_2_O) after MCAO surgical procedure. WT and KO mice in untreated groups were given vehicle alone.

### Neurological deficits

Neurological scores were evaluated a day after induction of transient focal cerebral ischemia (*n* = 6 per group). The scoring system used was as follows: 0, no observable neurological deficits (normal); 1, failure to extend the right forepaw (mild); 2, circling to the contralateral side (moderate); 3, falling to the right (severe); 4, inability to walk spontaneously; and 5, depressed level of consciousness (very severe). Each animal’s score was estimated within an approximate 1-min period and was estimated in quadruplicate for consistency. Score values > 0 were used to indicate the presence of behavioral deficits; a score of 0 corresponded to a normal neurological status.

### Measurement of infarct volume

A day after induction of transient focal cerebral ischemia, each mouse (*n* = 6 per group) was anesthetized using pentobarbital sodium and then euthanized. A mouse brain matrix (L.M.S. Korea, Seongnam-Si, South Korea, L.R-68713) was used to section the whole brains into 2-mm-thick coronal slices. The brain slices were then incubated in 2,3,5-triphenyltetrazolium chloride (TTC; 2%; Sigma-Aldrich, St. Louis, MO) for 20 min at 37 °C to reveal the ischemic infarction. After the TTC reaction was complete, ImageJ analysis software (version 1.6 NIH) was used to measure the cross-sectional areas of infarction and non-infarction for each section. The unstained areas indicated the presence of ischemic lesions. Infarct volume was calculated based on a 2-mm section thickness. Each side of the brain sections was measured separately, and mean values were calculated. Integration of four chosen sections was used to estimate the total infarct volume. Each result was expressed as a percentage of the total brain volume.

### Immunofluorescence staining

For immunofluorescence staining, each brain section was incubated (2 h) in blocking solution at room temperature. Each section was then incubated at 4 °C overnight with mouse anti-CD31 antibody (1:100; BD Pharmingen), rabbit anti-ZO-1 antibody (1:100; Invitrogen), rabbit anti-occludin antibody (1:100; Invitrogen), rabbit anti-claudin-5 (1:50; Invitrogen) antibody, mouse anti-glial fibrillary acidic protein antibody (GFAP, 1:1000; Millipore, Burlington, MA), rabbit anti-Iba1 antibody (1:200; Wako, Richmond, VA), rabbit anti-VCAM-1 antibody (1:100; Santa Cruz), or rabbit anti-ICAM-1 antibody (1:100; Santa Cruz). Each section was washed five times in Triton X-100 (0.1%) in PBS for 15 min, and then incubated overnight (4 °C) with secondary antibody. Before washing, each section was exposed to 1 μg/ml DAPI and washed five more times with 0.1% Triton X-100 (0.1%) in PBS for 10 min each. Each antibody was dissolved in antibody diluent (Dako, Santa Clara, CA). The confocal images were recorded at room temperature with ZEN software and an upright confocal microscope (LSM 700; Carl Zeiss) using predefined ZEN software configurations for Alexa Fluor 594, Alexa Fluor 488, and DAPI.

### Quantitative real-time reverse transcription polymerase chain reaction

Total RNA (isolated from WT and CLEC14A-KO mouse brain after ischemia-reperfusion injury) was reverse transcribed to cDNA and amplified with the primers on a PikoReal 96 Real-Time PCR System (Thermo Fisher Scientific) using Maxima SYBR Green/ROX qPCR Master Mix (Thermo Fisher Scientific; catalog K0221) for quantitative real-time reverse transcription polymerase chain reaction (qRT-PCR) assays. All qRT-PCR assays were performed in triplicate in at least three independent experiments using two different samples. The PCR primers are listed in Table 1.

**Table 1:** Primer sequences used for RT-PCR

Primer	Direction	Sequence
IL-6(M)	Fwd	5′-TACCACTTCACAAGTCGGAGGC-3’
	Rev	5′-CTGCAAGTGCATCATCGTTGTTC-3’
IL-1b(M)	Fwd	5′-TGGACCTTCCAGGATGAGGACA-3’
	Rev	5′-GTTCATCTCGGAGCCTGTAGTG-3’
TNF-a(M)	Fwd	5′-GACGTGGAACTGGCAGAAGA-3’
	Rev	5′-CCGCCTGGAGTTCTGGAA-3’
MCP-1(M)	Fwd	5′-CACTCACCTGCTGCTACTCA-3’
	Rev	5′-CTTCTTGGGGTCAGCACAGA-3’
GAPDH(M)	Fwd	5′-CATCACTGCCACCCAGAAGACTG-3’
	Rev	5′-ATGCCAGTGAGCTTCCCGTTCAG-3’

### Statistical analysis

The results were presented as mean ± standard deviation or mean ± standard error of the mean. GraphPad Prism software (version 5.0; GraphPad Software, La Jolla, CA) was used for all statistical analyses. ANOVA with post hoc Bonferroni’s multiple comparison tests was used to compare multiple group means. A *P* value of < 0.05 was considered to be statistically significant.

## Results

### CLEC14A deficiency increased endothelial permeability and decreased junctional integrity

**Fig. 1:**
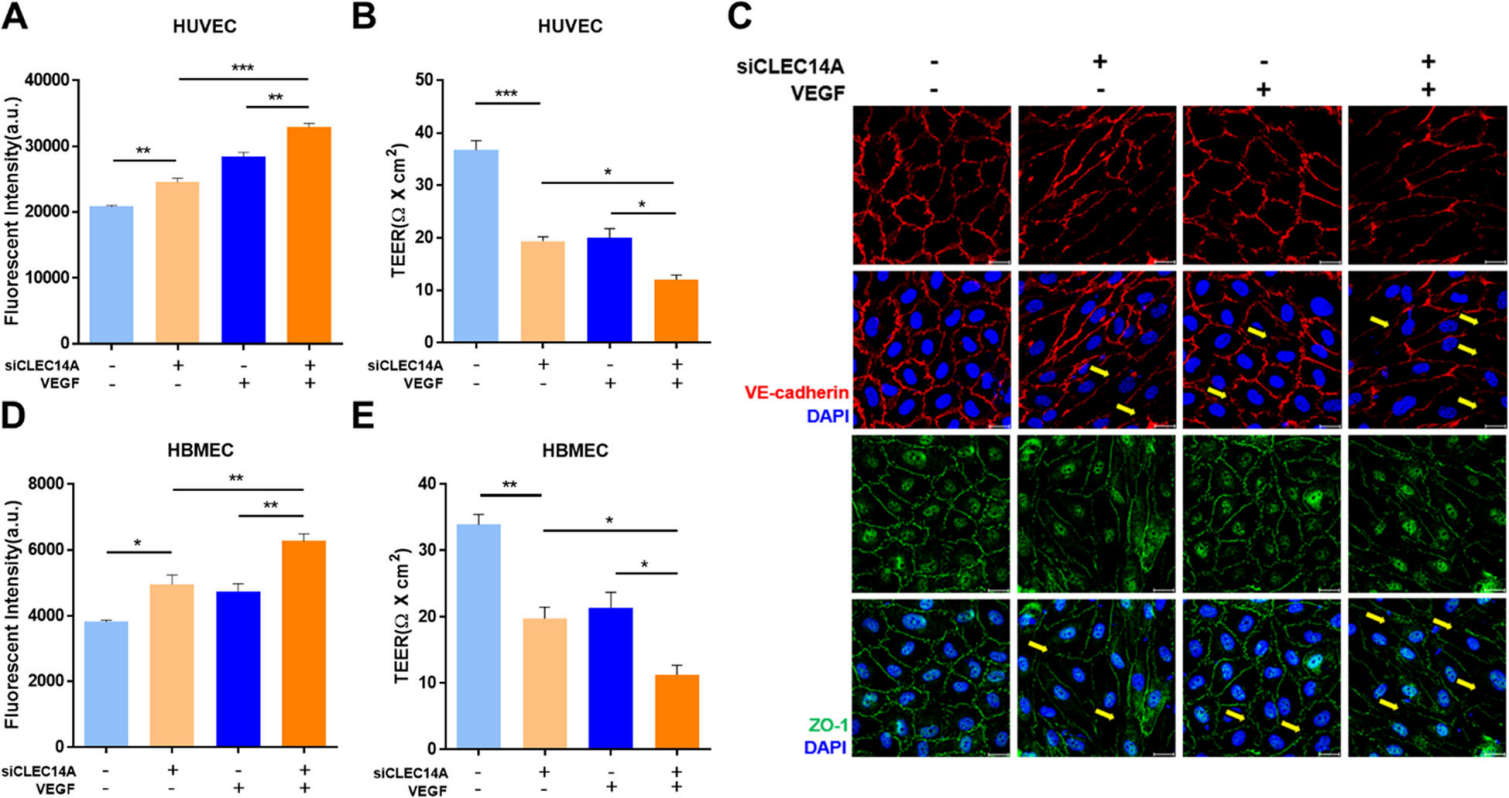
CLEC14A knockdown decreased junctional integrity in ECs. **a**, **d** HUVEC and HBMEC monolayer permeability to FITC-dextran increased after knockdown of CLEC14A using siRNA(50 nM) and treatment with VEGF (50 ng/ml, 30 min). Absorbance of the solution in the lower chamber was measured after FITC-dextran was added to the transwell. **b**, **e** TEER was reduced by VEGF stimulation under the same conditions of the permeability assay before FITC-dextran was added. The TEER was measured using a Millicell ERS-2 (Millipore). **c** Immunofluorescence staining of VE-cadherin, ZO-1, and DAPI in untreated or VEGF-treated (50 ng/ml, 30 min) HUVECs after knockdown of CLEC14A. Each arrow indicates an attenuated junction. Scale bars: 20 μm. *P < 0.05, **P < 0.01, and ***P < 0.001. The results are mean values and the error bars represent the standard error of the mean values

To identify the effects of CLEC14A deficiency that increase endothelial permeability, we performed in vitro vascular permeability assays using FITC-conjugated dextran (70 kD, FITC-dextran) and TEER. HUVECs were monolayer-cultured, and CLEC14A were silenced using siRNA. HUVEC monolayer permeability to FITC-dextran was increased and became severe after treatment with VEGF (Fig. 1a). TEER was reduced in CLEC14A knockdown HUVECs, and there was more TEER decline with VEGF stimulation (Fig. 1b). The vascular integrity was determined by examining junctional proteins. We tested the stability of the adherens junction protein, VE-cadherin, and the tight junctional protein ZO-1 using immunofluorescence staining. CLEC14A silencing significantly attenuated junctional protein expression in the VEGF-treated condition (Fig. 1c). We used HBMECs to confirm the role of CLEC14A in the BBB. HBMECs were cultured using the same conditions as the HUVECs, except for the culture media conditions. We measured FITC-dextran permeability and TEER. HBMECs also had increased endothelial permeability and decreased TEER (Fig. 1d, e). These results indicated that CLEC14A contributed to the stability of endothelial cell-to-cell junctions and vascular integrity.

### Increasing VEGFR-2 expression increased CLEC14A KO mouse brain injury in a cerebral transient focal ischemia model

**Fig. 2:**
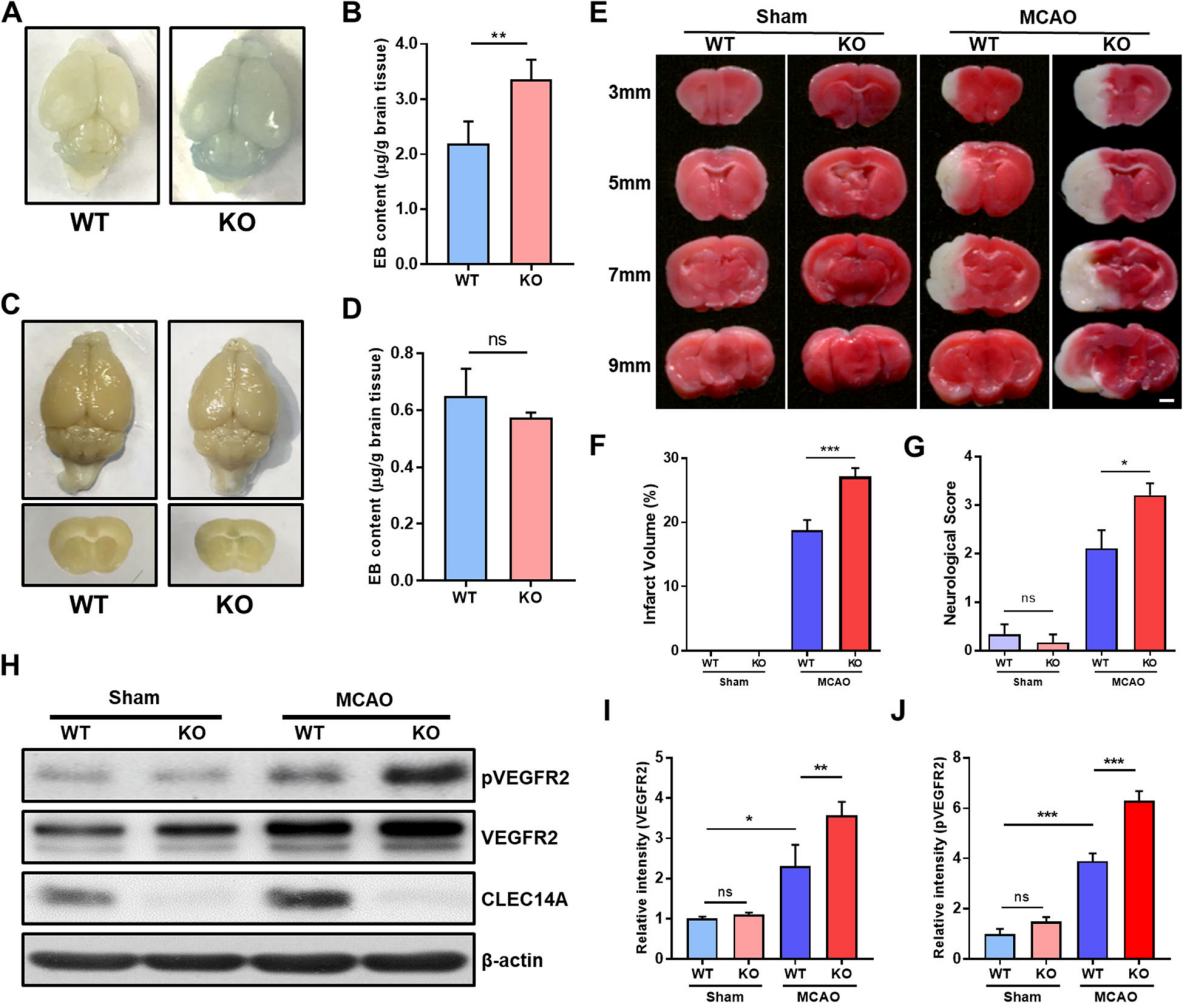
Loss of CLEC14A exacerbated cerebral injury with VEGFR2 activation in MCAO stroke model. **a**, **c** Images of whole brains and comparison of Evans blue dye (EB) leakage in brains (P8 and adult) after EB injection (intraperitoneal route) in WT and CLEC14A KO mice (*n* = 6, each group). **b**, **d** The quantitative analysis of Evans blue (EB) dye extravasation using spectrophotometer, 24 h after intraperitoneal injection of EB dye. **e** TTC staining of brain slices in the sham, WT, and CLEC14A KO groups 24-h post ischemia-reperfusion. Severe infarction was present in the CLEC14A KO group. Scale bar = 1 mm. **f** Percentage of infarct volume in the WT and CLEC14A KO groups 24 h after ischemia-reperfusion. **g** Neurological scores of WT and CLEC14A KO mice were evaluated. **h** VEGFR2, pVEGFR2 protein expression in ipsilateral brain lysate after ischemia-reperfusion injury. **i**, **j** Quantification data of relative pVEGFR2 and VEGFR2 protein expression. *n* = 6 per group; *P < 0.05, **P < 0.01, ***P < 0.001. The results are mean values and the error bars represent the mean ± SD

To evaluate whether vascular defects occurred in the brains of CLEC14A knockout mice, we used EB dye to reveal cerebral vascular leakage. EB dye was injected (intraperitoneal route) into postnatal day 8 mice; the mice were euthanized 1 day after injection. More vascular leakage was found in whole brains of CLEC14A KO mice than the wild-type (WT) littermates (Fig. 2a, b). However, brains of adult mice had no differences in BBB maturation and angiogenesis (Fig. 2c, d). The BBB permeability to a smaller tracer (FITC-dextran; 4 kD) was also evaluated, and there was no significant difference of FITC-dextran permeability in both WT and CLEC14A KO mice (see Additional file 1: Figure S1). Next, we used a focal cerebral ischemia model (MCAO) with WT and CLEC14A KO mice to examine the loss of CLEC14A and high expression of VEGFR-2 during the pathological condition. TTC staining was performed to evaluate infarct volume 24 h after focal cerebral ischemia-reperfusion. Infarcted regions were not detected in the sham groups. However, in the MCAO groups, the CLEC14A KO mice had severe infarction of the cerebral regions 24 h after ischemia-reperfusion, compared with the WT group (Fig. 2e, f). Neurological scores were evaluated to examine the relationship between infarct volume and neurological impairment. The CLEC14A KO mice had higher neurological scores than those of the WT mice (Fig. 2g). To support that these results were associated with vessel hyperpermeability caused by upregulated VEGFR-2 expression, we isolated whole brains and used lysates of ipsilateral hemispheres to examine protein expression after MCAO. In the sham group, the CLEC14A KO mice had slightly increased VEGFR-2 expression, but the phosphorylated VEGFR-2 level was not significantly changed in the CLEC14A KO mice, compared with the WT mice. However, both VEGFR-2 and phosphorylated VEGFR-2 expression were highly upregulated in the CLEC14A mice, compared with the WT mice, after ischemia-reperfusion injury (Fig. 2h–j). These results suggested that upregulated VEGFR-2 caused by CLEC14A deficiency exacerbated the cerebral damage from ischemic stroke.

### CLEC14A deletion in mice increased cerebral vascular leakage after ischemia-reperfusion injury

**Fig. 3:**
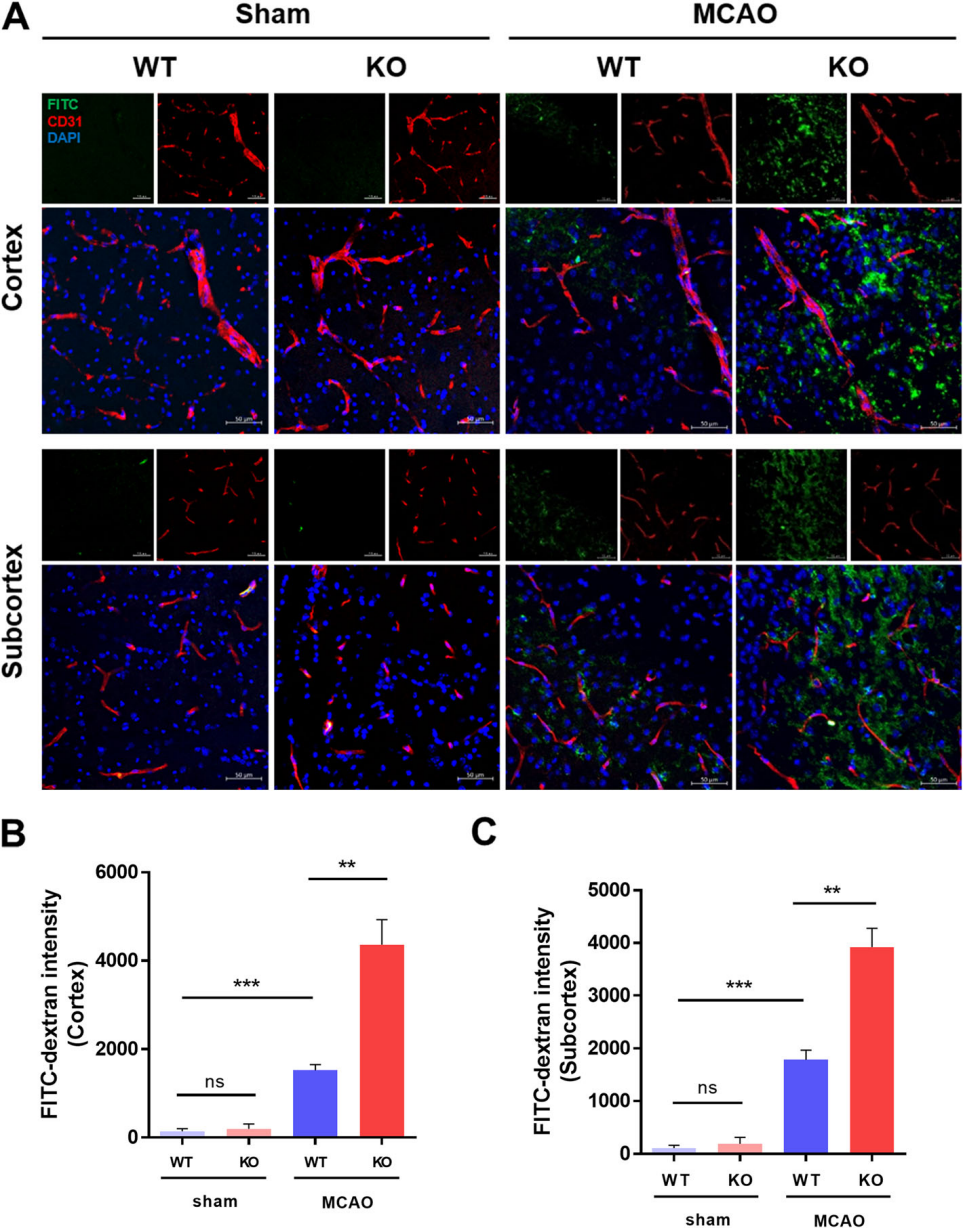
CLEC14A deletion increased cerebral vascular permeability after ischemia-reperfusion. **a** Confocal microscopic images of the cerebral cortex and subcortex. FITC-dextran (70 kD, 30 mg/ml) was injected in left ventricle 24 h after ischemia-reperfusion. *n* = 6 per group. Scale bars: 50 μm. **b**, **c** Quantitative analysis was performed on values for FITC-dextran mean intensity of cerebral cortex and subcortex in WT and CLEC14A KO mice. **P < 0.01, ***P < 0.001. The results are mean values and the error bars represent the mean ± SD

To test the effects of increased BBB permeability in CLEC14A KO mice after ischemia-reperfusion, FITC-dextran was injected (transcardial) 24 h after ischemia-reperfusion. Frozen sectioned tissues and immunofluorescence staining of cluster of differentiation 31 (CD31) were used to evaluate leakage of FITC-dextran. The leakage was more intense in the ischemic cerebral cortex of CLEC14A KO mice, compared with WT mice (Fig. 3a, b). This increased leakage was also found in the ischemic cerebral subcortex (Fig. 3a, c). It has been shown that cerebral ischemia induces opening of the BBB as early as 2 h after stroke [22], so we evaluated the BBB permeability and cerebral injury at earlier time-points. The CLEC14A KO mice developed severe cerebral injury at 2 and 6 h after ischemic stroke and showed more EB dye leakage than WT mice (see Additional file 1: Figure S2A and B).

**Fig. 4:**
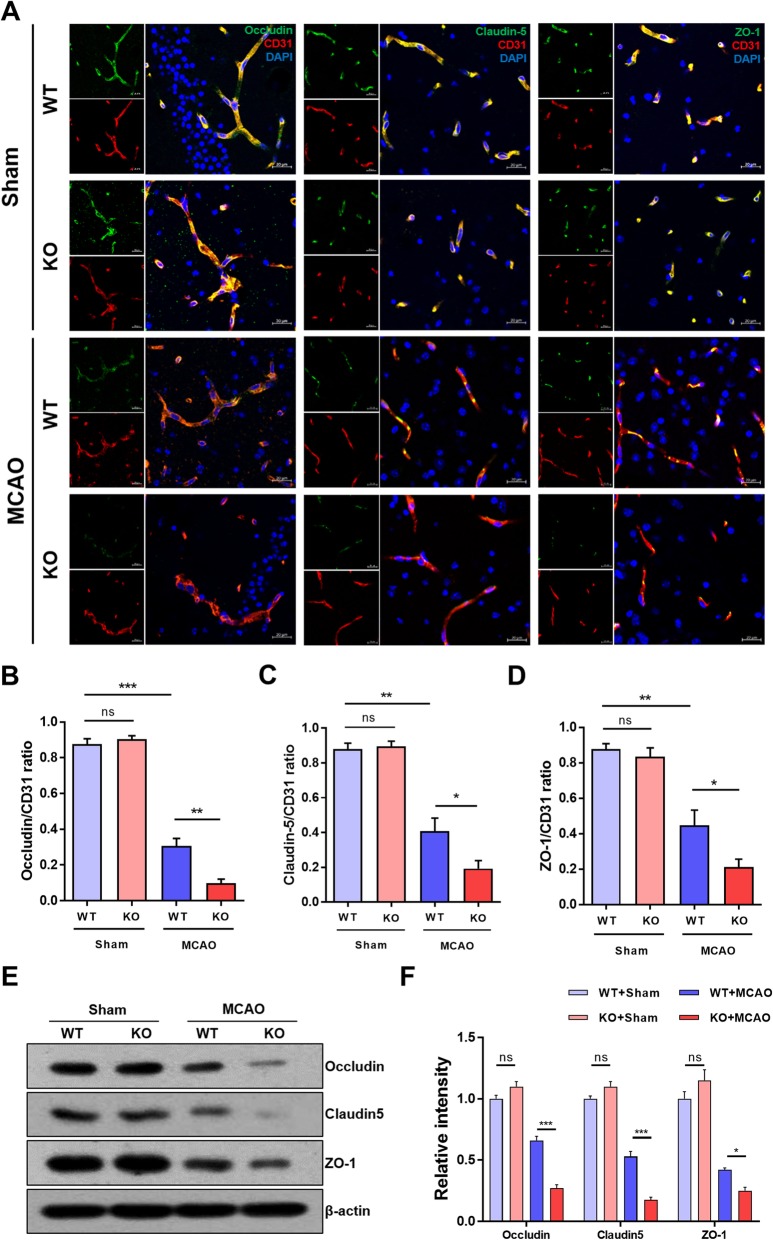
CLEC14A deletion attenuated tight junctional proteins in cerebral ischemic regions. **a** Immunofluorescence staining for Occludin, Claudin-5, ZO-1, and CD31 with DAPI in the ischemic cortex of WT and CLEC14A KO mice 24 h after ischemia-reperfusion. *n* = 6 per group. Scale bars: 20 μm. **b** Quantitative analysis of Occludin/CD31 ratio. **c** Quantitative analysis of Claudin-5/CD31 ratio. **d** Quantitative analysis of ZO-1/CD31 ratio. **e**, **f** Western blot analysis of tight junctional proteins from ischemic brain and quantitative graph. *P < 0.05, **P < 0.01, and ***P < 0.001. The results are mean values and the error bars represent the mean ± SD

Our results support the hypothesis that loss of CLEC14A increases cerebral vascular permeability because of the attenuation in junction-related proteins that occurs after ischemic stroke. In the central nervous system, the tight junctions of cerebral ECs form a barrier to restrict paracellular permeability. The tight junctional molecules Occludin, Claudin-5, and ZO-1 were immunofluorescence-stained with endothelial marker CD31. After ischemia-reperfusion injury, Occludin, Claudin-5, and ZO-1 intensity remained relatively strong in the WT group (Fig. 4a). In contrast, expression of tight junctional proteins was significantly reduced in the CLEC14A KO group. The results for the quantification of the data from the confocal microscopic images are presented in Fig. 4b–d. We further tested the protein expression of tight junctions of the ischemic hemisphere using Western blot analysis and found that CLEC14A-KO mice had significantly decreased expression of these molecules compared with WT mice (Fig. 4e, f). CLEC14A deletion attenuated the BBB via decreased endothelial tight junction expression and increased cerebral vascular leakage after ischemia-reperfusion.

### CLEC14A KO mice had an increased inflammatory response caused by expression of adhesion molecules and pro-inflammatory cytokines after ischemic stroke

**Fig. 5:**
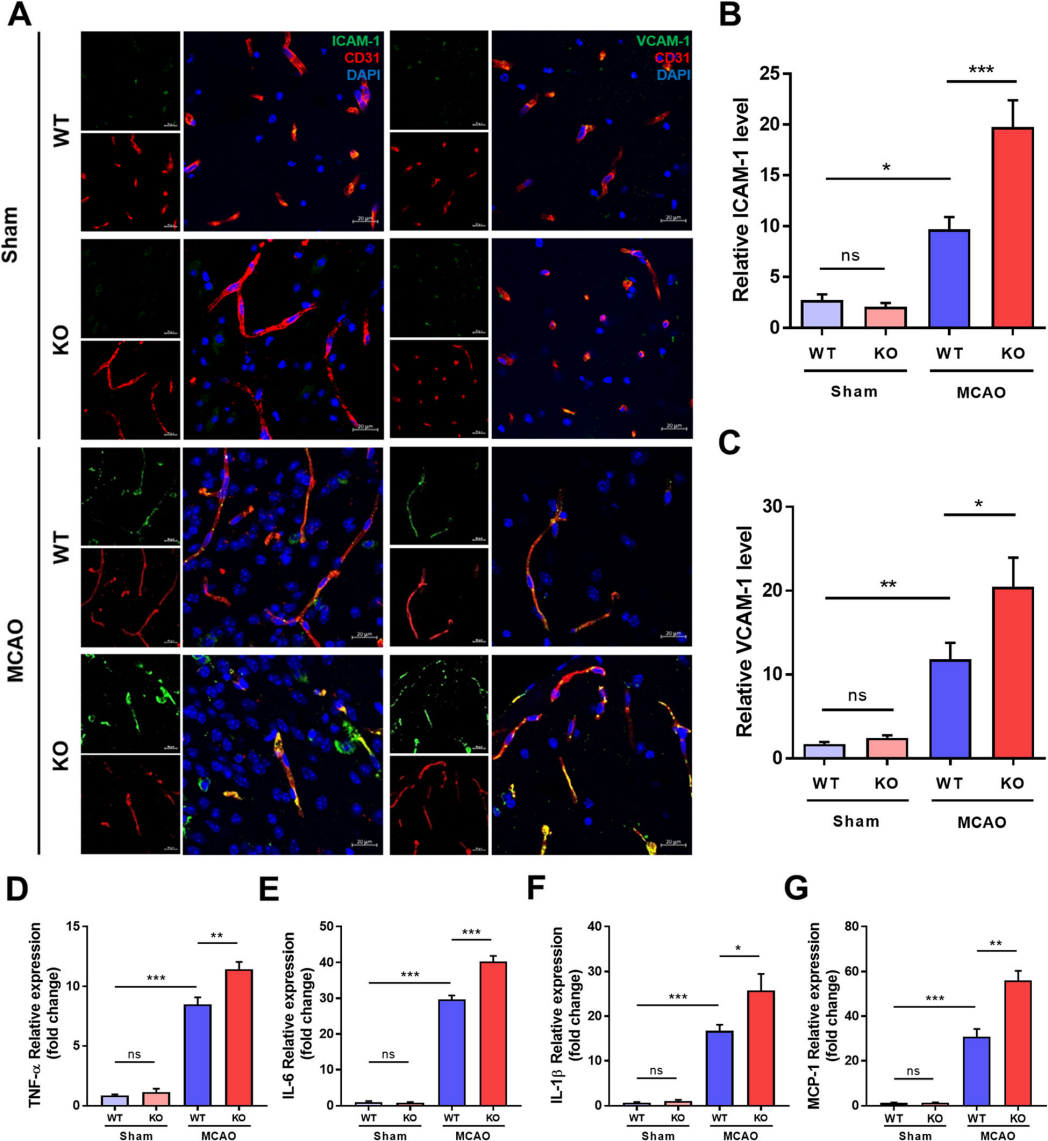
Loss of CLEC14A upregulated expression of adhesion molecules and pro-inflammatory cytokines after ischemia-reperfusion. **a** Representative images of results for ICAM-1 and VCAM-1 immunofluorescence staining in the ischemic region of WT and CLEC14A KO mice. Merged images of ICAM-1 or VCAM-1 with CD31 and DAPI staining are also shown. *n* = 6 per group. Scale bars: 20 μm. **b**, **c** Quantification of the relative ICAM-1 or VCAM-1 levels. **d**–**g** Relative mRNA expression level of TNF-α, IL-6, IL-1β, and MCP-1. *P < 0.05, **P < 0.01, and ***P < 0.001. The results are mean values and the error bars represent the mean ± SD

The endothelial cell adhesion molecules ICAM-1 and VCAM-1 are important for mediation of tissue injury during ischemic stroke. In brain disorders, the BBB becomes impaired and many inflammatory factors are upregulated. These factors include adhesion molecules that bind to leukocyte ligands and allow migration of leukocytes into brain tissue. Evaluation of the inflammatory response using immunofluorescence staining of adhesion molecules revealed BBB breakdown after the MCAO surgical procedure. In the sham groups, WT and KO mice had little expression of ICAM-1 and VCAM-1. However, in the MCAO group, CLEC14A KO mice had higher levels of expression of adhesion molecules in the cerebral ischemic regions after ischemia-reperfusion injury than WT mice (Fig. 5a–c). To further support the effect of CLEC14A deficiency on pro-inflammatory mediators, we confirmed the change of mRNA expression by qRT-PCR. Pro-inflammatory cytokines, such as tumor necrosis factor alpha (TNF-α), interleukin 6 (IL-6), interleukin 1 beta (IL-1β), and monocyte chemoattractant protein-1 (MCP-1), were significantly increased in the CLEC14A-KO mice after stroke (Fig. 5d–g). These results suggest that the inflammatory response was increased by BBB disruption through the high VEGFR-2 expression associated with the pathological condition in CLEC14A KO mice.

### Activation of glial cells increased in CLEC14A KO mice after ischemia-reperfusion injury

**Fig. 6:**
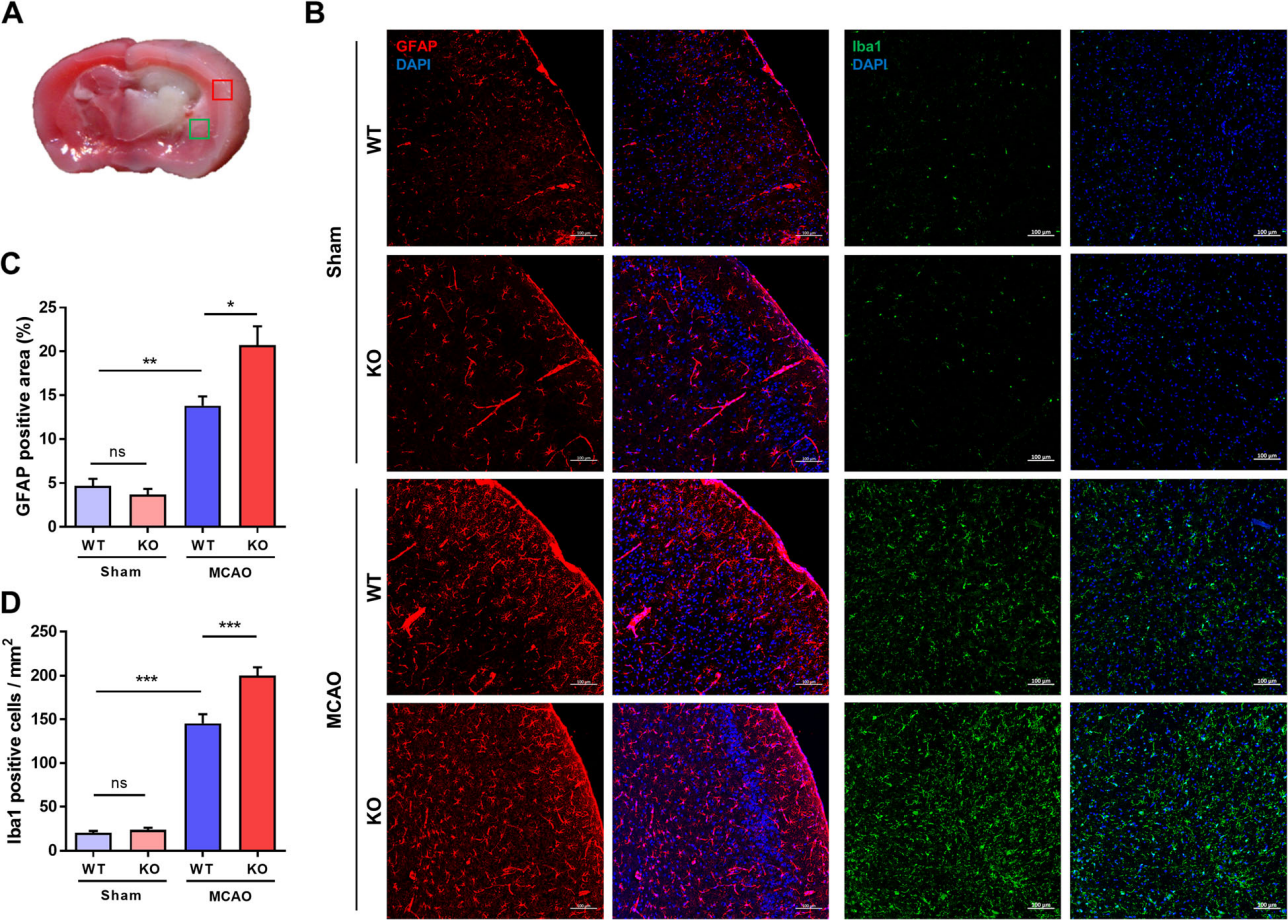
CLEC14A KO mice exhibited glial activation after ischemia-reperfusion. **a** Picture of TTC stained coronal brain section indicating immunofluorescence staining regions. The square fields represent the observed regions of GFAP (red) and Iba1 (green). **b** Immunofluorescence staining of GFAP (red) and Iba1 (green) in the ischemic cortex of the WT and CLEC14A KO groups after ischemia-reperfusion. Increased expression of GFAP and Iba1 was present in the CLEC14A-KO group, compared with the WT group. *n* = 6 per group. Scale bars: 100 μm. **c**, **d** Glial activation was quantified based on the percentage of the GFAP-positive area and Iba1 positive cells per mm^2^. *P < 0.05, **P < 0.01, and ***P < 0.001, paired comparisons. The results are mean values and the error bars represent the mean ± SD

Perivascular cells (e.g., astrocytes and microglia) have an important role in the neuro-inflammatory response induced by cerebral ischemic stroke. Astrocytes are the major population of glial cells and maintain brain homeostasis. We used immunofluorescence staining and observed activation of GFAP-positive astrocytes in the ischemic region to investigate differences in activated astrocytes in the cerebral ischemic cortex after ischemia-reperfusion. Significantly more activated astrocytes were found in the CLEC14A KO mice after ischemic stroke, compared with the WT mice (Fig. 6b, c). Microglia fulfill the pro-inflammatory response to cerebral injury by migrating to and reacting at the injury site. Like astrocytes, the Iba1-positive microglial cells were more increased in the cerebral infarct region of the CLEC14A KO mice than the same region of the WT group, after ischemia-reperfusion injury (Fig. 6b, d). Taken together, these results indicated that loss of CLEC14A induced a primary immune response by upregulated adhesion molecules and reactive glial cells during ischemic stroke conditions.

## Discussion

Stroke is the leading cause of death and disability worldwide. BBB breakdown is a pathological hallmark of stroke, but the mechanisms of this process are unclear. We found that hyperactivation of VEGFR-2 induced BBB dysfunction in an MCAO model using CLEC14A KO mice. Study results suggest that CLEC14A is a crucial regulator of physiological and pathological responses [20, 23]. However, the functional significance of CLEC14A in cerebrovascular disorders remains unknown.

In this study, we found that CLEC14A deficiency increased endothelial leakage. This result was supported by our experimental evidence indicating that FITC-dextran permeability was increased and TEER was decreased in HUVECs and HBMECs after VEGF-A treatment following CLEC14A knockdown using siRNA. Our results also indicated that deletion of CLEC14A suppressed junctional protein expression. Use of EB dye extravasation revealed that the CLEC14A KO mice had cerebral vascular leakage early in the postnatal period, compared with the WT littermates. However, there were no between-group differences in adult mice. These results correspond to the results of our previous study that indicated that CLEC14A KO mice were viable and fertile. Taken together, these results indicate the presence of compensatory mechanisms for CLEC14A loss, despite hemorrhage during embryonic development [20].

To extend our previous results, we used a mouse model of cerebral transient focal ischemia. Administration of VEGF to the ischemic brain increased the size of the infarcted region and the amount of BBB leakage after focal cerebral ischemia [9, 24]. CLEC14A KO mice had larger areas of cerebral infarction and severe neurological defects after ischemia-reperfusion injury. This result was likely due to hyperactivation of VEGFR-2 during pathological angiogenesis in the CLEC14A KO mice. This analysis was supported by the results of the Western blotting analysis of ipsilateral hemisphere lysates from the infarcted brains. In the CLEC14A KO group, VEGFR-2 and phosphorylated VEGFR-2 expression was increased in infarcted brains. In our MCAO model using CLEC14A KO mice, FITC-dextran extravasation was easily observed in ischemic brains. We also evaluated BBB dysfunction using immunofluorescence staining of tight junctional proteins (e.g., claudin-5, occludin, and ZO-1). These results are consistent with the results of our previous study on developmental and tumoral angiogenesis, which indicate that decreased endothelial pericyte and smooth muscle cell coverage also occurs, indicating an increase in abnormal vessels [20]. Pericyte-deficient mice have reduced expression of BBB tight junctional proteins [25–27]. Our study found that tight junctional proteins were significantly decreased in the peri-infarct regions of CLEC14A KO mice after ischemia-reperfusion injury. These results suggest that reduced tight junctional proteins induced BBB leakage in CLEC14A KO mice with hyperactivation of VEGFR-2 signaling after ischemic stroke.

Ischemic stroke is accompanied by an increased inflammatory response as well as increased BBB permeability [28]. In the ischemic brain, inflammatory mediators stimulate cerebral ECs to a pro-inflammatory response [29]. Microglia remain in a deactivated phenotype in physiological conditions. They are activated in response to pathogen invasion or cerebral damage and subsequently increase the inflammatory response [30, 31]. Expression of adhesion molecules (e.g., ICAM-1 and VCAM-1) is mediated by activation of the NF-κB pathway, which is typically studied as an indicator of inflammation in models of cerebral ischemia [32, 33]. The results of our MCAO model using CLEC14A-KO mice indicated that expression of ICAM-1 and VCAM-1 was increased after ischemia-reperfusion, compared with WT mice. The main pro-inflammatory cytokines belong to the IL-1 families, IL-6, TNF families, and MCP-1 [34, 35]. These cytokines are initiators of the inflammatory response and promote the expression of adhesion molecules [36, 37]. Our results support that these pro-inflammatory mediators upregulated adhesion molecules and worsened the outcome of stroke in CLEC14A-KO mice. As components of the BBB structure, glial cells maintain the integrity and function of this barrier [38]. Study results suggest that activated glial cells correspond to neuronal loss and endothelial impairment [39, 40]. In the peri-infarct regions of the ischemic brains, GFAP-positive astrocytes and Iba1-positive microglia were more activated in the CLEC14A KO mice. We found that an upregulated pro-inflammatory response and more activated glial cells were induced by the greater vascular permeability in the cerebral ischemic regions of CLEC14A KO mice after ischemia-reperfusion.

To strengthen our results, considering the effects of CLEC14A deletion on VEGFR-2 expression, we evaluated whether administration of the VEGFR-2 inhibitor (Semaxinib, SU5416) would rescue the BBB permeability and brain injury in the CLEC14A-KO mice after ischemia-reperfusion injury. SU5416, a well-known VEGFR-2 inhibitor, has a half-life of 72 h and can cross the BBB [41, 42]. Therefore, a single dose of SU5416 via subcutaneous injection is sufficient to significantly reduce BBB permeability induced by stroke [43]. In our study, the cerebral infarct was rescued by treatment with SU5416 in CLEC14A KO mice after ischemic stroke. In addition, EB extravasation was also decreased (see Additional file 1: Figure S3A and B). The change of tight junctional protein expression, pro-inflammatory response, and glial activation needs to be confirmed through more detailed experiments. However, our results suggest a correlation between hyperactivation of VEGFR2 and BBB disruption in CLEC14A-KO mice after ischemic stroke.

Numerous studies show that angiogenesis is highly important for a neuroprotective effect, and the formation of new blood vessels is regarded as a potential therapeutic target in ischemic stroke [44–47]. However, the acute phase of increasing vessels after a stroke had negative effects, like BBB leakage [47]. As a consequence, impaired angiogenesis increases vascular permeability, brain edema, and infarct volume [48, 49]. Our previous study showed that CLEC14A KO mice developed abnormal vessels, so impaired angiogenesis due to CLEC14A deficiency might also have an effect on stroke. Further research is needed to understand the mechanisms of CLEC14A in angiogenesis after stroke and to determine if CLEC14A might be useful as a therapeutic target.

## Conclusions

VEGF-A and its receptor, VEGFR-2, have been found to be essential for the treatment of stroke via angiogenesis, neuroprotection, and neurogenesis. However, our results show that hyperactivated VEGFR-2 signaling in CLEC14A KO mice exacerbated BBB leakage and inflammatory response, with increased pro-inflammatory cytokines and glial activation induced by ischemic stroke. Therefore, our findings suggest that appropriate VEGF-A/VEGFR-2 signaling is important for the treatment of cerebral damage after ischemic stroke in the CLEC14A-KO mice.

## Supplementary information

**Additional file 1:Figure S1.** BBB permeability of adult mice brain in WT and CLEC14-KO mice. Figure S2. Evaluation of the BBB permeability and brain injury at earlier time-points after MCAO. Figure S3. Cerebral injury and BBB leakage were rescued by administration of VEGFR-2 inhibitor, SU5416.

## Data Availability

Not applicable.

## Abbreviations

BBB: Blood-brain barrier
CD31: Cluster of differentiation 31
CLEC14A: C-type lectin family 14 member A
EB: Evans blue
FITC: Fluorescein isothiocyanate
GFAP: Glial fibrillary acidic protein
HBMEC: Human brain microvascular endothelial cell
HUVEC: Human umbilical vein endothelial cell
Iba1: Ionized calcium-binding adapter molecule 1
ICAM-1: Intercellular adhesion molecules-1
IL-1β: Interleukin-1 beta
IL-6: Interleukin-6
KO: Knockout
MCAO: Middle cerebral artery occlusion
MCP-1: Monocyte chemoattractant protein-1
NF-ĸB: Nuclear factor-kappa B
PBS: Phosphate buffer solution
TEER: Transendothelial electrical resistance
TNF-α: Tumor necrosis factor alpha
VCAM-1: Vascular adhesion molecule-1
VEGF: Vascular endothelial growth factor
VEGFR: Vascular endothelial growth factor receptor
ZO-1: Zonula occludens-1
