# Supplementary material for: CLEC14A deficiency exacerbates neuronal loss by increasing blood-brain barrier permeability and inflammation
Source: J Neuroinflammation. 2020 Feb 4;17:48. doi: 10.1186/s12974-020-1727-6 (PMC7001304; doi:10.1186/s12974-020-1727-6)
Supplement: Supplementary file 1 — Additional file 1:Figure S1. BBB permeability of adult mice brain in WT and CLEC14-KO mice. Figure S2. Evaluation of the BBB permeability and brain injury at earlier time-points after MCAO. Figure S3. Cerebral injury and BBB leakage were rescued by administration of VEGFR-2 inhibitor, SU5416. [file 12974_2020_1727_MOESM1_ESM.pdf]

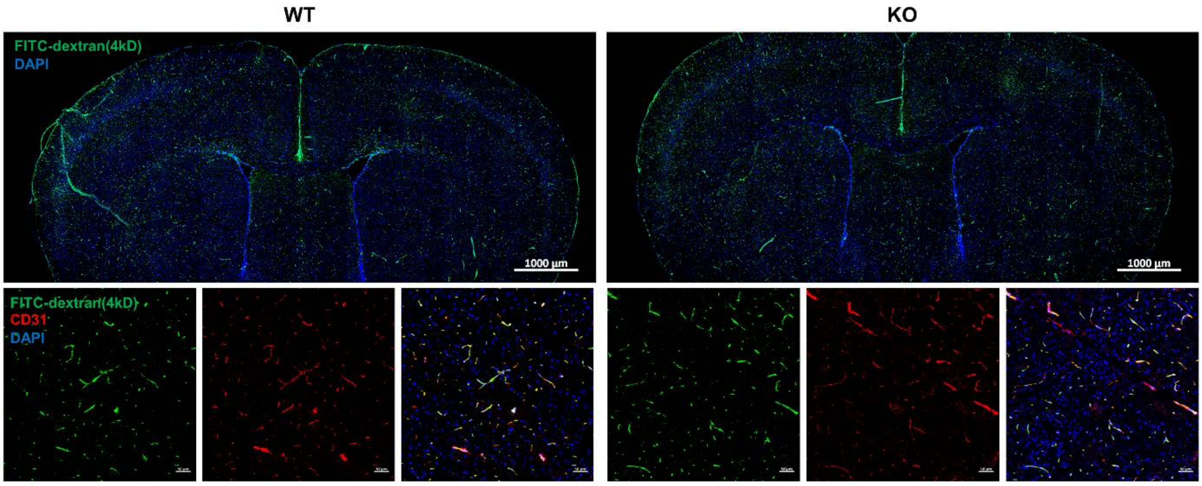

Figure S1. BBB permeability of adult mice brain in WT and CLEC14-KO mice

Confocal microscopic images of adult mice brain. The BBB permeability was evaluated with FITC-dextran (4 kD) and counterstained with DAPI (upper pannel). Scale bars: 1,000 µm. Immunofluorescence staining of CD31 with DAPI in the cerebral cortex injected FITC-dextran (lower pannel). Scale bars: 50 µm.

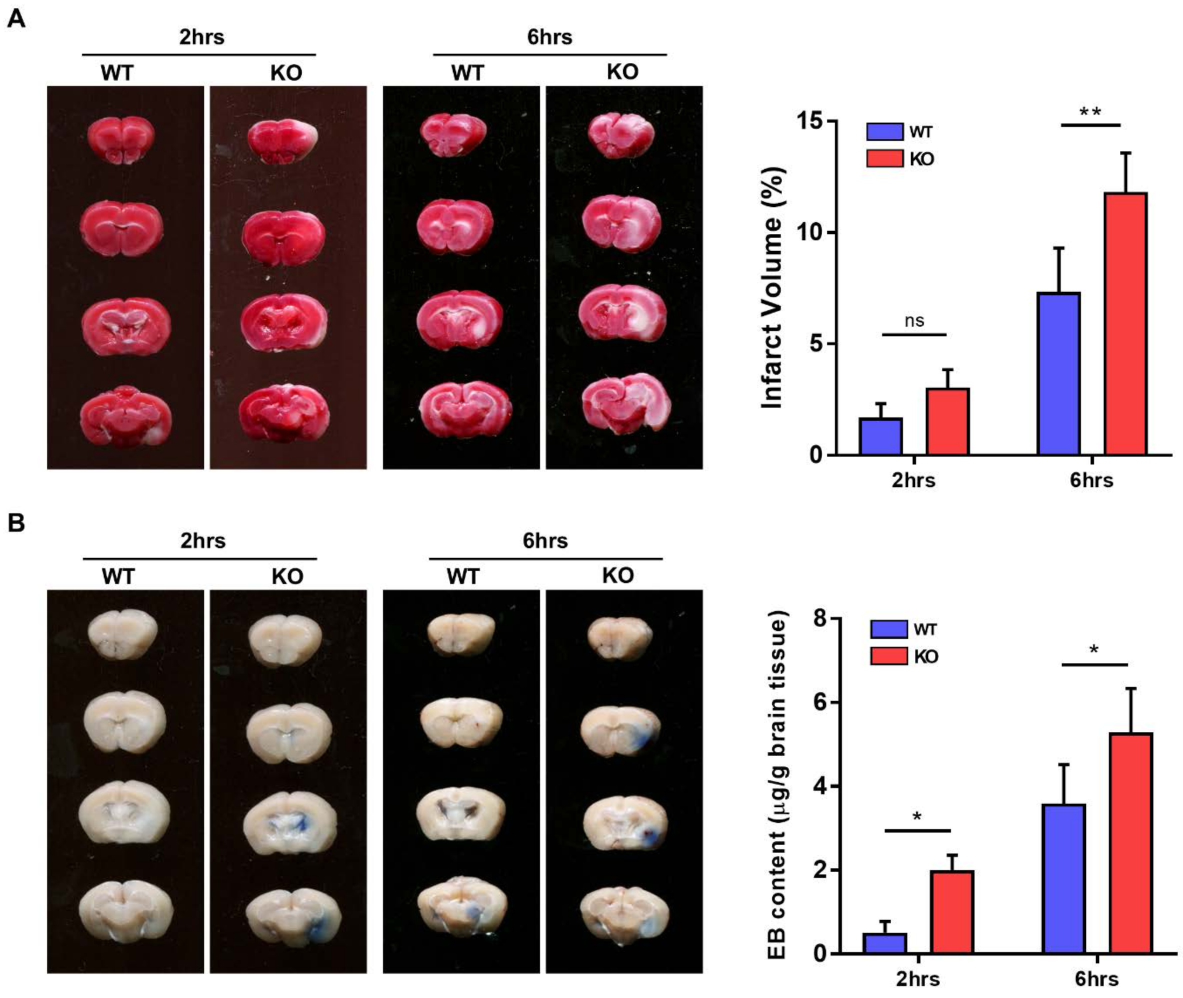

Figure S2. Evaluation of the BBB permeability and brain injury at earlier time-points after MCAO.

(A) TTC staining of brain sections in WT and KO mice 2 h and 6 h after stroke. Quantitative analysis of infarct volume was also shown. n = 5 per group. **(B)** Images of EB leakage in brain slices and quantitative graph. Evans blue dye was injected (intravenous) 30 minutes before sacrifice. n = 4-5 per group. *$P < 0.05$, **$P < 0.01$. The results are mean values and the error bars represent the mean ± SD.

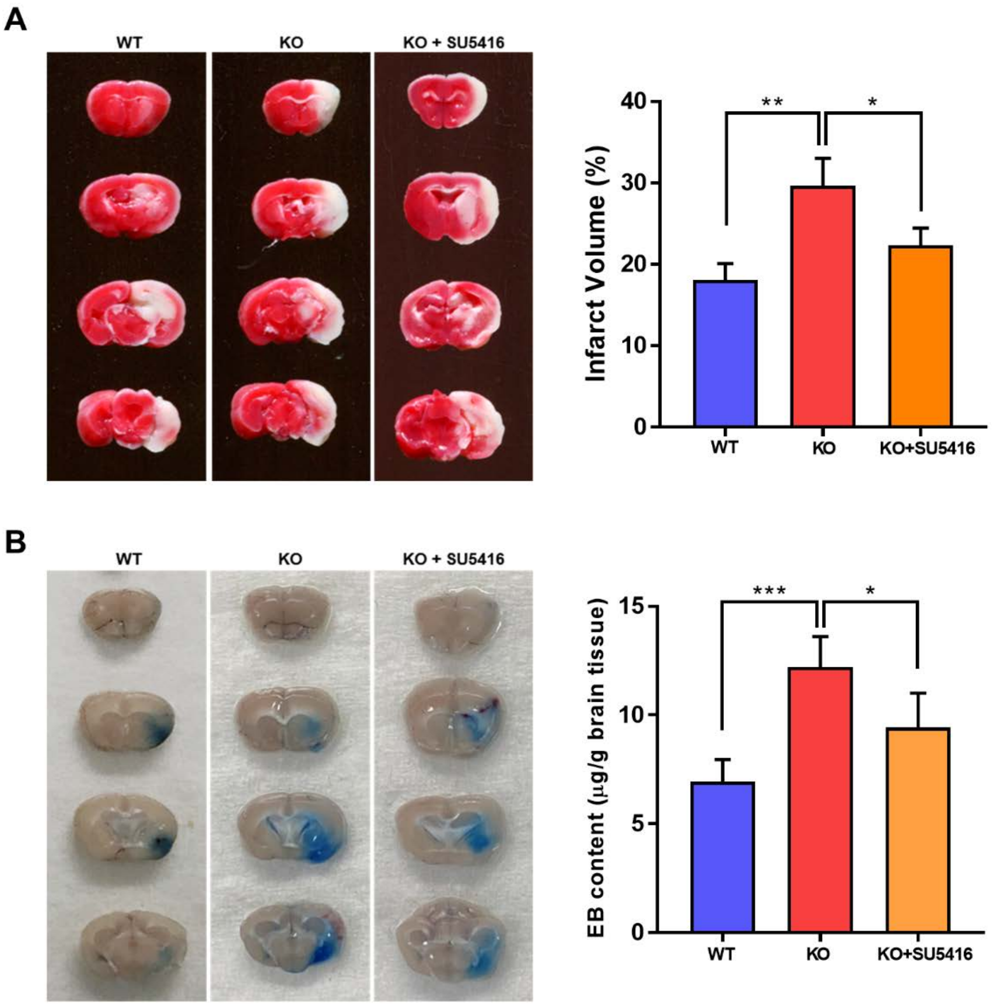

Figure S3. Cerebral injury and BBB leakage were rescued by administration of VEGFR-2 inhibitor, SU5416.

(A) Representative images of TTC stained brain slices and quantitative data. Mice in untreated groups were given vehicle alone. n = 4-6 per group. **(B)** Images of coronal brain slices with EB dye injection after ischemia-reperfusion injury and quantitative analysis of EB dye extravasation. n = 4-5 per group. *P < 0.05, **P < 0.01, and ***P < 0.001. The results are mean values and the error bars represent the mean ± SD.
